# 1729. Literature Review to Identify Evidence of Secondary Transmission of ROTATEQ® (RV5) Vaccine Strains to Unvaccinated Subjects

**DOI:** 10.1093/ofid/ofad500.1561

**Published:** 2023-11-27

**Authors:** Yuanqiu Li, Xiaojin Sun, Yaqun Fu, Xuedan You, Susanne Hartwig

**Affiliations:** MSD Research and Development (China) Co.,Ltd., Beijing, China, Beijing, Beijing, China; MSD Research and Development (China) Co.,Ltd., Beijing, China, Beijing, Beijing, China; MSD Research and Development (China) Co.,Ltd., Beijing, China, Beijing, Beijing, China; Merck & Co., Inc.,, Rahway, New Jersey; MSD Vaccins, Lyon, Rhone-Alpes, France

## Abstract

**Background:**

Rotavirus (RV) is the leading cause of severe diarrhea in infants and young children. RV vaccination is recommended by WHO since 2009 for all children worldwide, especially in countries with a high number of diarrhea-associated deaths. Live attenuated vaccines can lead to horizontal transmission with the risk of vaccine-derived disease in contacts. Transmission of RV5 strains leading to clinical disease was not well evaluated in the pivotal clinical trials and only few case-reports have been described in the literature.

**Methods:**

We performed a systematic literature review to investigate secondary transmission of RV5 vaccine strains to unvaccinated subjects globally. We searched Embase, Medline for English papers, CNKI, Wan Fang for Chinese papers and other resources (i.e., conference papers with full text, preprint platform including bioRxiv and medRxiv) from January 2005 to June 2021. Eligibility criteria for inclusion were original articles bases on non-interventional studies (case-control studies, cohort studies, cross-sectional studies) using RV5 vaccine strain transmission as outcomes. Other study or publication types were excluded such as pre-clinical studies, interventional studies and case reports. A Preferred Reporting Items for Systematic Reviews and Meta-Analyses (PRISMA) was used and study quality was assessed using the Newcastle Ottawa Scale (NOS) for cohort studies and the JBI checklist for cross-sectional studies to assess risk of bias.

**Figure:**
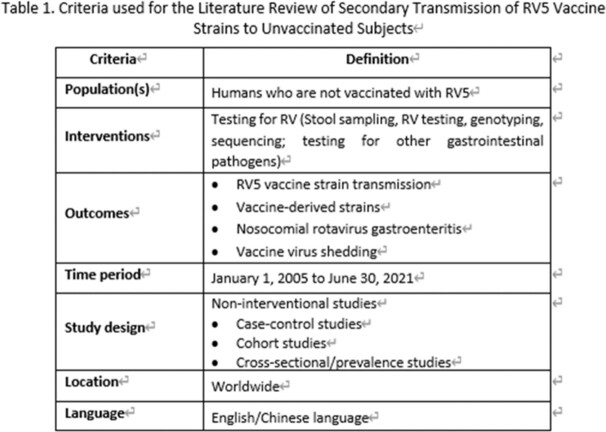

**Figure:**
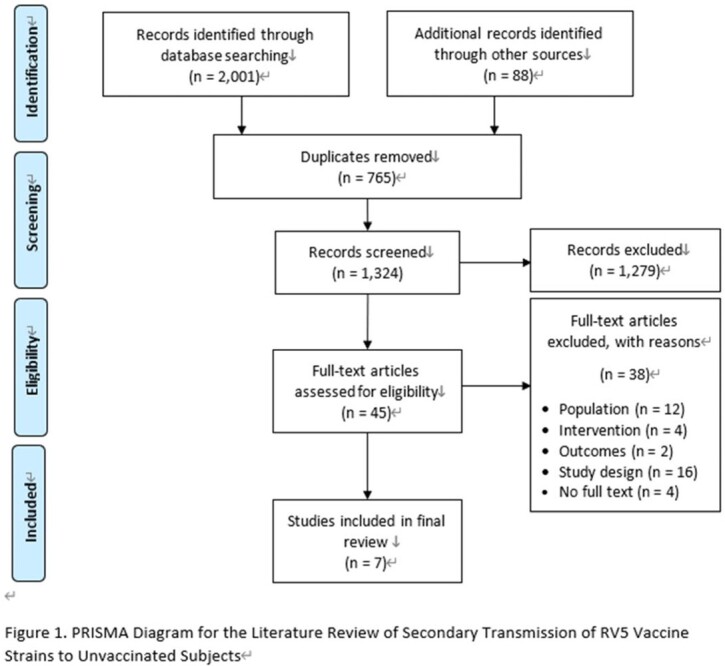

**Results:**

The search generated 2,089 articles in total. Seven articles met all inclusion criteria, including six cohort studies and one cross-sectional study. All studies underwent quality assessment and complied with the quality criteria of NOS or JBI checklist respectively. Overall, none of the seven studies identified RV5 vaccine-type transmission to an unvaccinated population, in either hospitals or nurseries under a close contact environment. One study reported that 1% of unvaccinated infants had gastrointestinal symptoms but all symptoms were attributed to other clinical conditions.

**Figure:**
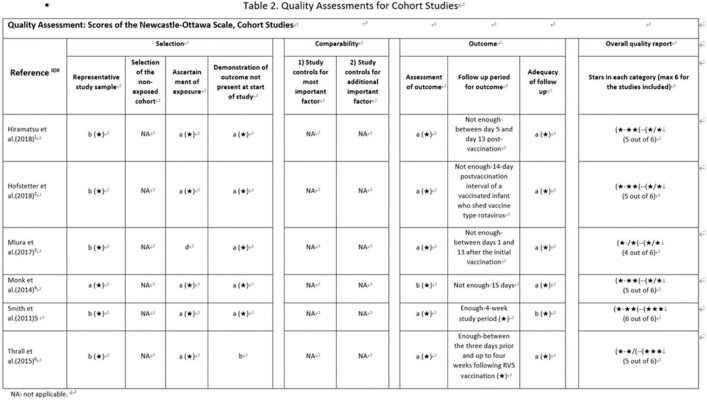

**Figure:**
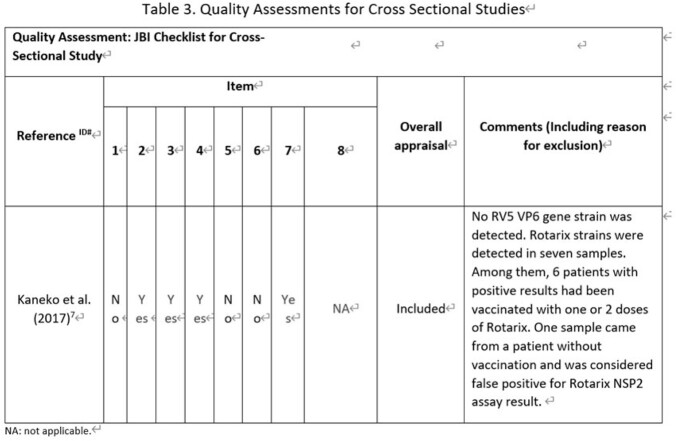

**Figure:**
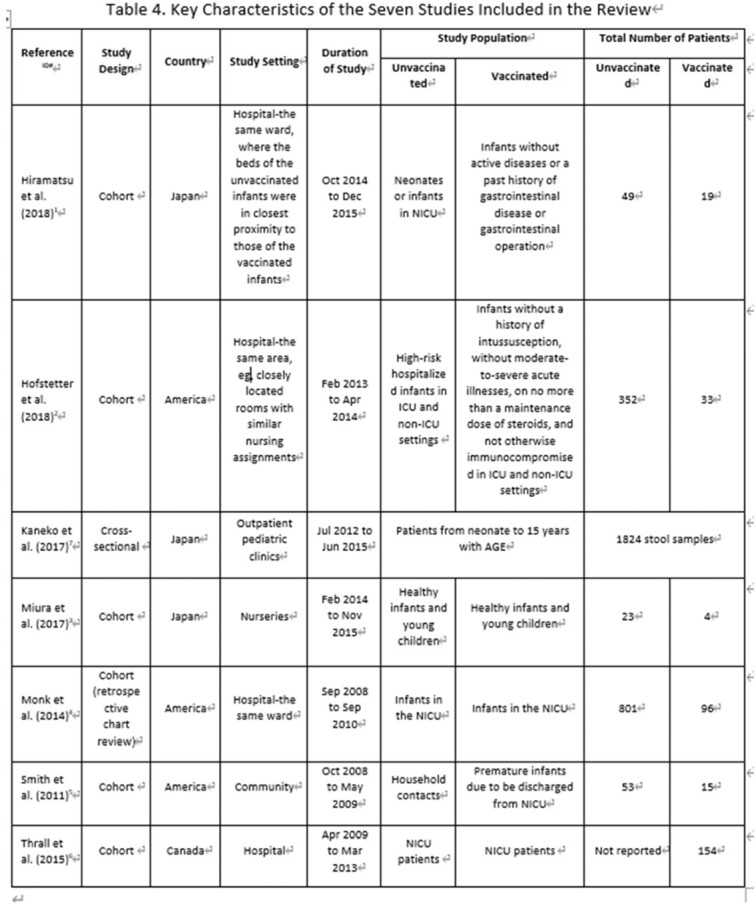

**Conclusion:**

We found no evidence of horizontal transmission of RV5 vaccine virus strains to unvaccinated infants despite the limited amount and descriptive nature of the identified studies.

**Figure:**
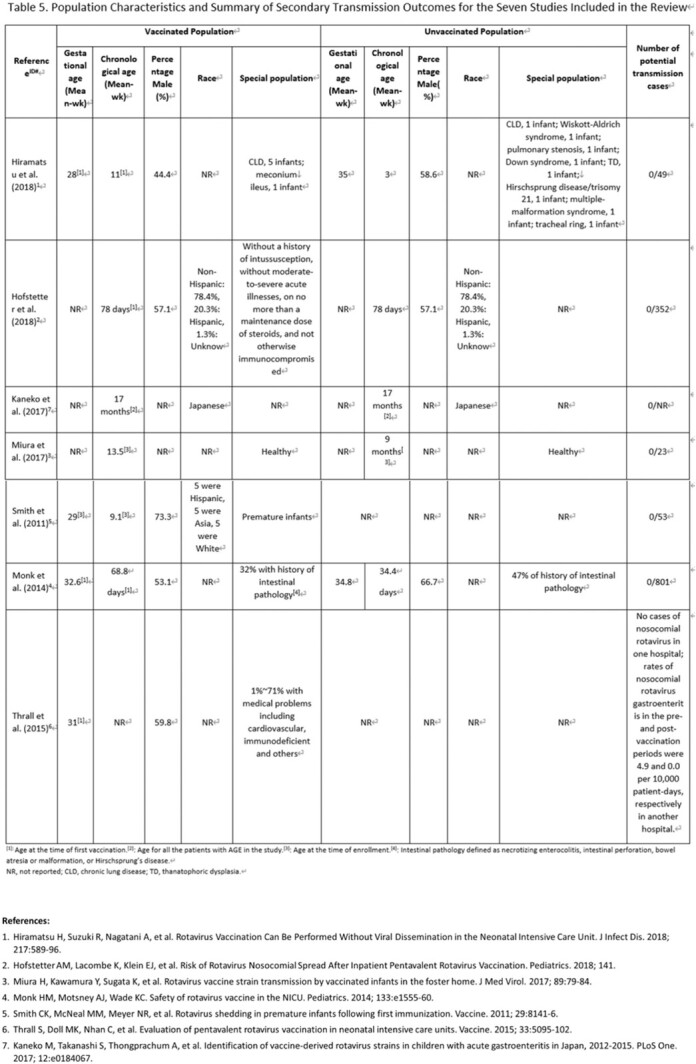

**Disclosures:**

**All Authors**: No reported disclosures

